# Variable cellular decision-making behavior in a constant synthetic network topology

**DOI:** 10.1186/s12859-019-2866-6

**Published:** 2019-05-14

**Authors:** Najaf A. Shah, Casim A. Sarkar

**Affiliations:** 1 Genomics and Computational Biology Graduate Group, Perelman School of Medicine, University of Pennsylvania, Philadelphia, PA USA; 2 Department of Biomedical Engineering, College of Science and Engineering, University of Minnesota, Minneapolis, MN USA

**Keywords:** Topology, Design reuse, Decision-making, Multi-modality, Gene dosage

## Abstract

**Background:**

Modules of interacting components arranged in specific network topologies have evolved to perform a diverse array of cellular functions. For a network with a constant topological structure, its function within a cell may still be tuned by changing the number of instances of a particular component (e.g., gene copy number) or by modulating the intrinsic biochemical properties of a component (e.g., binding strength or catalytic efficiency). How such perturbations affect cellular response dynamics remains poorly understood. Here, we explored these effects in a common decision-making motif, cross-antagonism with autoregulation, by synthetically constructing this network in yeast.

**Results:**

We employed the engineering design strategy of reuse to build this topology with a single protein building block, TetR, creating necessary components through TetR mutations and fusion partners. We then studied the impact of several topology-preserving perturbations – strength of cross-antagonism, number of operator sites in a promoter, and gene dosage – on decision-making behavior. We found that reducing TetR repression strength, which hinders cross-antagonism, resulted in a loss of mutually exclusive cell responses. Unexpectedly, increasing the number of operator sites also impeded decision-making exclusivity, which may be a consequence of the averaging effect that arises when multiple transcriptional activators and repressors are accommodated at a given locus. Stochastic simulations of this topology revealed that, even for networks with high TetR repression strength and a low number of operator sites, increasing gene dosage can reduce exclusivity in response dynamics. We further demonstrated this result experimentally by quantifying gene copy numbers in selected yeast clones with differing phenotypic responses.

**Conclusions:**

Our study illustrates how parameters that do not change the topological structure of a decision-making network can nonetheless exert significant influence on its response dynamics. These findings should further inform the study of native motifs, including the effects of topology-preserving mutations, and the robust engineering of synthetic networks.

**Electronic supplementary material:**

The online version of this article (10.1186/s12859-019-2866-6) contains supplementary material, which is available to authorized users.

## Background

Systems biology studies have identified numerous molecular networks that govern diverse cellular processes such as stem cell differentiation and the cell cycle [1]. The arrangement of molecular components according to a particular architecture can considerably constrain its dynamic behavior and thereby lend robustness to its function. For instance, a network with stimulus-driven receptor activation and downstream transcriptional feedback can be more easily tuned to exhibit ultrasensitivity to the stimulus, than if the network lacked transcriptional feedback [2, 3]. Analogously, networks that exhibit adaptation to a stimulus generally adhere to one of two simple topologies [4]. Despite constraints imposed by the architecture, however, two instances of the same network topology can exhibit qualitatively different behaviors. In fact, this represents a core challenge in implementing synthetic systems, requiring iterative modification and testing to implement the desired functionality [5].

Starting with a biological system with a given topology, its behavior can be modified in two key ways: by adjusting the intrinsic properties of its components (e.g., changing the operator binding affinity of a transcription factor or the catalytic efficiency of an enzyme) or by modulating the quantities of its components (e.g., changing the number of operator sites or the basal concentration of an enzyme). Understanding how such modifications yield qualitative differences in behavior can not only enable us to construct synthetic circuits with less iteration, but can also elucidate how such changes can lead to disease. Towards this goal, we constructed a series of synthetic circuits in *Saccharomyces cerevisiae* that share the same underlying network topology, but vary in their gene dosage, number of transcription factor binding sites, and/or repression strength.

**Fig. 1:**
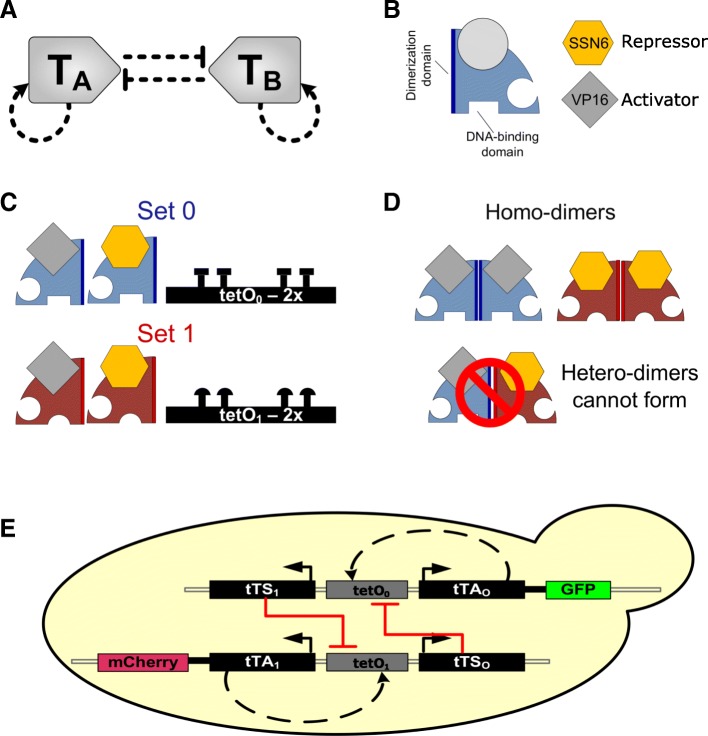
Synthetic implementation of the CAA circuit. **a** The CAA topology consists of two auto-regulating, mutually-inhibiting transcription factors (*T*_*A*_, *T*_*B*_). **b** Schematic for the TetR monomer which comprises dimerization, drug-binding, and DNA-binding domains. **c** Mutations in the DNA-binding and dimerization domains enable the simultaneous, non-interfering use of an altered-specificity TetR (Set 1) along with the original TetR (Set 0). **d** Dimerization is possible only between monomers of the same set. **e** Circuit implementing the CAA topology. The CAA model comprises two opposing sides, *T*_*A*_, *T*_*B*_; here, *T*_*A*_ is represented by the transcriptional activator tTA_0_, the repressor tTS_0_, and the promoter tetO_0_. Similarly, TB is represented by the transcriptional activator tTA_1_, the repressor tTS_0_, and the promoter tetO_1_. The activators are fused to either GFP or mCherry to allow for tracking via fluorescence measurements. The two sides are delivered on separate plasmids that integrate into yeast chromosomes

Numerous studies utilizing systems modeling have proposed the cross-antagonism with autoregulation (CAA) network topology to drive decision-making behavior in various contexts [6–9]. The CAA topology consists of a pair of transcription factors, each upregulating its own synthesis, and repressing the other’s synthesis (Fig. 1a). This system can yield three steady states: two in which one transcription factor is present in high abundance and the other is present in low abundance, and, if it exists, a third state in which both are present in intermediate abundances [10–12]. If the transcription factors drive separate expression programs (e.g., for different lineages or decisions), then the CAA topology in effect enables the cell to decide between these programs. Taken further, chaining of multiple CAA modules together in a hierarchy presents an elegant theoretical mechanism by which a cell can decide among an array of fates [13].

Modeling studies of the CAA network have yielded several important predictions. First, the CAA network alone is sufficient to yield non-genetic multi-modality, with each cell randomly choosing one of multiple fates. Second, strong mutual repression is essential for exclusive expression of either transcription factor, and in turn, its expression program. Third, the distribution of cells among the three states can be modulated by adjusting the relative strengths of transcription activation and repression [10, 12]. These predictions have not been directly addressed by experimental studies.

Using the engineering design strategy of reuse [14], which advocates assembling complex systems by using multiple instances of robust building blocks, we constructed a CAA circuit in the yeast *Saccharomyces cerevisiae* composed of mutants and fusions of a single core protein. Our circuit offers the following advantages: the core protein building block, tetracycline repressor (TetR) [15], is not from yeast, so the study of the CAA circuit in this non-native environment should reduce undesired host interactions; the modularity of the system enables focused genetic perturbations including modulation of repression strength, promoter architecture, and gene dosage via plasmid copy number; and the fusion of fluorescent reporters to the opposing transcriptional activators enables real-time tracking of ‘cell fate.’

The response dynamics and decision-making exclusivity of this synthetic CAA network were then probed through a number of perturbations that do not alter the topology: modulating the strength of transcriptional repression, changing the number of operator sites, and altering gene dosage. A reduction in repression strength hindered the emergence of exclusive cell fates due to limited cross-antagonism. Increasing the number of operator sites also led to loss of exclusivity, likely due to the population averaging effects that arise when multiple activators and repressors can be simultaneously recruited to the same locus. As predicted by stochastic simulations and validated experimentally with individual CAA-containing clones, increasing the number of gene copies hinders exclusive decision-making. Our study reveals the significant roles that promoter architecture and gene dosage can play in modulating the dynamic behavior of a network without fundamentally changing its topological structure.

## Results

### Reuse of TetR variants

The abstract CAA network topology consists of two transcription factors, *T*_*A*_ and *T*_*B*_, each promoting its own synthesis and inhibiting the synthesis of the other. In our network, *T*_*A*_ and *T*_*B*_ are each represented with an activator-repressor pair, so the implemented network is composed of four proteins with four topological connections (Fig. 1a). However, each protein is, at its core, the same base TetR protein; depending on the required function, this protein is mutated to change its DNA operator sequence and/or dimerization specificities, and fused with a transcriptional activation or repression domain.

Rational design, directed evolution, and genotyping experiments have revealed a series of TetR variants [15]. Mutating three amino acids in TetR (V36F, E37A, P39K) changes its DNA-binding specificity to a different operator sequence, tetO-4C5G. Importantly, the newly identified DNA-binding domain and tetO-4C5G operator sequence pair is specific to the canonical DNA-binding domain and operator sequence, tetO, meaning that each pair should not significantly interfere with the other [16]. This suggests the possibility of using the two variants to regulate two separate sets of genes within the same cell. However, since TetR proteins form dimers, simultaneous expression of both variants would lead to a significant number of heterodimers. This can, in turn, lead to an unpredictable combination of sequestration (since one variant can prevent the other from performing its function) and aberrant actuation (since only one monomer in the heterodimer would bind to half of the operator sequence, it is unclear what the overall DNA-binding on- and off-rates would be). To prevent heterodimerization, a separate dimerization specificity is needed.

To create a TetR variant with DNA-binding and dimerization specificities different from wild-type TetR, we made seven substitutions: three in the DNA-binding domain (noted above) and an additional four in the dimerization domain (F188H, L192S, I193L, L197F), as previously described [17]. We will refer to the dimerization domain, operator sequence, and DNA-binding domain combination for the canonical TetR as set 0 (denoted in subscript) and the variant combination as set 1 (Fig. 1b, c, d).

The CAA circuit requires pairs of transcriptional activators and repressors. We fused the VP16 activation domain [18] separately to both TetR_0_ and TetR_1_, yielding transcriptional activators, tTA_0_ and tTA_1_, with different operator and dimerization specificities. To enable tracking of transcription factor levels via microscopy and flow cytometry, tTA_0_ was additionally fused to GFP and tTA_1_ to mCherry.

Since TetR by itself exerts transcriptional repression, TetR_0_ and TetR_1_ can be used directly as the two repressors in the CAA circuit; in addition to these repressors, we constructed another set by fusing the strong SSN6 repression domain from yeast [19] to the C-termini of TetR_0_ and TetR_1_, yielding tTS_0_ and tTS_1_. We refer to repressors comprising TetR alone or TetR-SSN6 fusions as tTS, using weak or strong, respectively, to identify the specific protein.

The canonical method for gene regulation by TetR involves placement of the tetO operator sequence, which consists of tandem repeats of a 19-bp sequence, upstream of the gene of interest. In the literature, both two (tetO-2x) and seven (tetO-7x) repeats of the base tetO sequence have been described [20]. TetR is used to either activate or suppress the transcription of a target gene; hence, tetO-7x is used when a stronger effect is desired. However, since our circuit involves simultaneous activation and repression at given operator sites, the effect of operator site number on cellular decision-making is unclear a priori. Therefore, we constructed two different versions of each, yielding four distinct full promoter sequences: tetO_0_-2x, tetO_0_-7x, tetO_1_-2x, and tetO_1_-7x.

### Circuit construction and transformation

Having defined a complete parts list based on the design strategy of reuse, we translated the CAA wiring map into a gene regulatory network (Fig. 1e). The full circuit is arranged as follows. The transcriptional activator for set 0, tTA_0_, is regulated by a synthetic promoter containing a defined number of tetO_0_ operator sites. Basal transcription and subsequent translation leads to production of the tTA_0_ protein; in the absence of tetracycline, this protein can bind to the tetO_0_ sequences and hence promote its own synthesis in a positive feedback loop. Analogous inclusion of tetO_1_ operator sites in the promoter before the tTA_1_ gene creates a positive feedback loop for the other variant.

Transcriptional repression is implemented by placing the tTS_1_ gene under control of a synthetic promoter containing tetO_0_ operator sites and, analogously, the tTS_0_ gene under control of a synthetic promoter containing tetO_1_ operator sites. This arrangement accomplishes two tasks. First, placement of the tTS genes downstream of operator-specific sequences couples their expression to the relevant activators: tTS_1_ is transcribed as part of the tTA_0_-mediated feedback loop and tTS_0_ is transcribed as part of the tTA_1_-mediated feedback loop. Second, a tTS_0_ dimer can bind to the tetO_0_-containing promoter and suppress activation exerted by the tTA_0_-mediated feedback loop, and the tTS_1_ protein dimer can bind to the tetO_1_-containing promoter and suppress activation exerted by the tTA_1_-mediated feedback loop. Taken together, the circuit is composed of two opposing sides, representing *T*_*A*_ and *T*_*B*_ in the minimal CAA model. *T*_*A*_ consists of tTA_0_, tTS_1_, and tetO_0_, while *T*_*B*_ consists of tTA_1_, tTS_0_, and tetO_1_ (Fig. 1e).

To enable convenient, modular testing of different component combinations, we used a bi-directional promoter architecture. The full circuit is delivered on two plasmids, with each half encoded on one plasmid. Starting with the tetO operator site(s), we placed a minimal CYC1 promoter (containing a TATA box) at the 3′ end. Next, we added the tTA gene to the 3′ end of the CYC1 promoter, and a CYC1 terminator at the 3′ end of the tTA gene. We then placed another copy of the CYC1 promoter at the 5′ end of the tetO operator site(s). Next, we added the tTS gene to the 5′ end of this second CYC1 promoter. Finally, another copy of the CYC1 terminator was added to the 5′ end of the tTS gene.

Circuit genes were cloned into separate, chromosomally integrating plasmids with tTA_0_ and tTS_1_ on one plasmid, and tTA_1_ and tTS_0_ on another (details described in Methods). Simultaneous transformation of both plasmids into yeast cells yields colonies on selective medium. Cells within each colony are genotypically identical; however, the genotype of two colonies resulting from the same transformation reaction can be different, because each of the two plasmids can integrate in one or more copies. This difference in gene dosage can potentially yield a difference in the dynamic behavior or phenotype of the circuit.

### Synthetic CAA circuit can yield discrete decisions, with clones exhibiting a diverse spectrum of behaviors

**Fig. 2:**
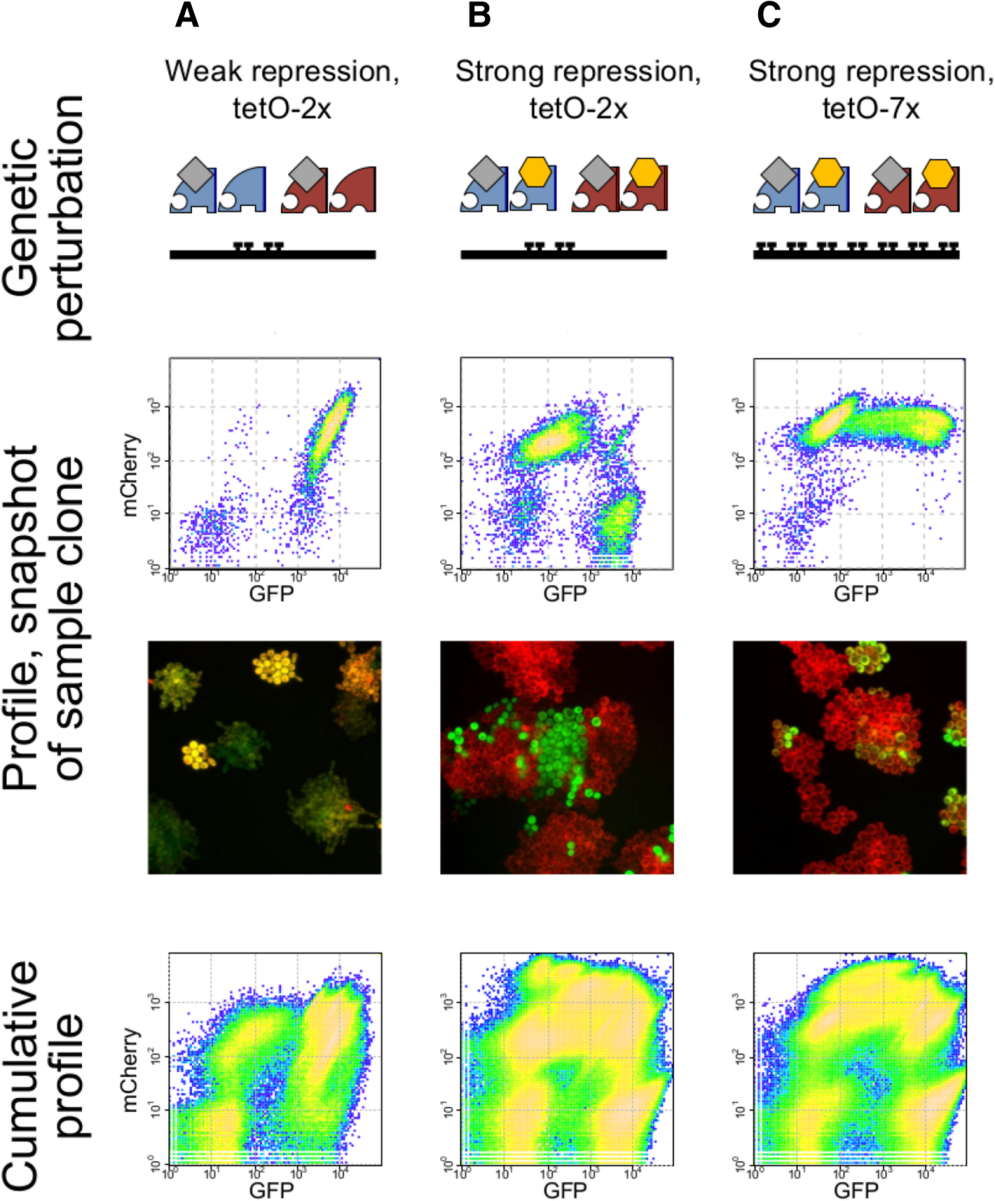
CAA circuit can generate a spectrum of behaviors and requires strong repression for discrete decisions. Comparison of behaviors observed under three different genetic perturbations. Only clones with the tetO-2x strong repression circuit are able to yield discrete decisions, with cells expressing high levels of either GFP or mCherry, but not both. Images and flow cytometry data captured at the 16-h time-point. Flow cytometry data from individual clones of each circuit variant were merged to create cumulative response profiles. **a** Weak repression for 2x tet operator sites. **b** Strong repression for 2x tet operator sites.** c** Strong repression for 7x tet operator sites

After obtaining transformant clones for the different variants of the synthetic CAA circuit (Fig. 2, top row), we performed a global survey of circuit behavior. For each circuit variant, several individual clones were separately grown in selective liquid medium, first supplemented with doxycycline to inhibit circuit expression until cells reached the exponential growth phase, and subsequently without doxycycline to allow circuit expression.

Analysis of expression revealed that for approximately the first 4 h after the removal of doxycycline, there is no significant expression of GFP or mCherry over background. Subsequently, fluorescence continues to rise in most clones, becoming very strong by the 16-h timepoint. Density plots of GFP and mCherry from flow cytometry data at the 16-h timepoint reveal two important points (Fig. 2, middle and bottom rows; Additional file 2: Video S1 and Additional file 3: Video S2). First, across different circuit variants and clones, cells within most individual cultures are tightly clustered into four discrete regions in phase-space: (high GFP, low mCherry), (low GFP, high mCherry), (high GFP, high mCherry), and (low GFP, low mCherry). In the context of the CAA model, clustering of cells by their GFP and mCherry expression levels within a genotypically identical population indicates the presence of multiple stable states, and demonstrates that the CAA circuit can yield discrete decisions. Second, despite clustering of cells into discrete regions, clones from the same transformation reaction yield a spectrum of GFP and mCherry response profiles that differ in two aspects: the placement of the four discrete states in GFP-mCherry space, and the distribution of the clonal population among these states. We hypothesized that this response-profile diversity among clones from the same transformation reaction is attributable to chance differences in plasmid copy numbers, which we examined later.

First, we analyzed in detail the responses of CAA topologies with altered repression strength and/or operator architecture (Fig. 2) to understand the effects of these specific perturbations on system dynamics.

### Strong mutual repression is a requirement for exclusive states

Our previous simulations and analysis of the CAA network topology indicate that the strength of repression exerted by *T*_*A*_ and *T*_*B*_ is a key determinant of the placement of the bipotent state in (*T*_*A*_, *T*_*B*_)-space and the relative proportions of cells committing to the different states [10, 21]. Furthermore, if repression strength is sufficiently weakened, the two mutually exclusive states (high *T*_*A*_, low *T*_*B*_) and (low *T*_*A*_, high *T*_*B*_) are eliminated, rendering the overall system monostable [10, 21].

We evaluated these predictions experimentally by comparing two variants of our synthetic circuits: one with weak repression activity conferred by occlusion of the promoter via binding of TetR and another with strong repression achieved by the SSN6 domain. Unlike TetR alone, TetR fused to SSN6 can potentially exert multiple types of repressive activity at the promoter, by interfering with the mediator complex to halt transcription by RNA polymerase II and by recruiting histone de-acetylation machinery to inactivate the promoter [22]. Both circuit variants examined include the 2x version of the tetO operators.

Comparison of GFP and mCherry expression profiles reveals the following. First, none of the clones of the weak-repression circuit examined exhibit both the (high GFP, low mCherry) and the (low GFP, high mCherry) exclusive states (Additional file 1: Figure S1). In contrast, a large number of clones from the strong-repression circuit variant yield both exclusive discrete states (Additional file 1: Figure S2). Second, in most profiles of the weak-repression set, the distribution of cells is heavily skewed to the (high GFP, high mCherry) state. Taken together, our experimental results support the CAA model prediction that weakening of repression strength can lead to the population being biased towards the (high GFP, high mCherry) state, and even to the abolishment of the exclusive states as the system becomes monostable.

### Multiple operator sites impede exclusivity in decision-making

In the context of stem-cell lineage commitment, the CAA topology models the behavior of a bipotent progenitor, with the (high *T*_*A*_, low *T*_*B*_) and (low *T*_*A*_, high *T*_*B*_) states representing committed, mature cell lineages [10]. After commitment, the mature cell must express its own program, and suppress the other lineage’s program that defines its identity. In order to yield this exclusivity in decision-making, the system must be parameterized such that in the bipotent state, expression of both transcription factors is sufficiently low to prevent aberrant induction of their gene expression programs. In mathematical terms, the bipotent (medium *T*_*A*_, *T*_*B*_) state should be closer to the origin than to the (high *T*_*A*_, high *T*_*B*_) point on the phase plot [10, 21].

Our experimental analysis of expression profiles across different circuit variants and clones reveals that, in general, strong exclusivity is rare. This implies that the balance between the two opposing sides is crucial, and is difficult to optimize in implemented circuits. How natural systems achieve nearly digital behavior through precise gene-regulation amidst the constant binding and un-binding of transcription factors and other components remains unclear.

To investigate this question further, we modified the operator sites in the strong repression, tetO-2x circuit and constructed a version with tetO-7x operators. At a genetic level, this circuit contains five additional tetO operator sites, yielding substantially increased opportunities for both the repressor and activator proteins to bind and exert their effects. Comparison of response profiles from these two strong-repression circuit variants reveals that, at a global level, the contrasts between high and low states for both GFP and mCherry are higher in the tetO-7x set and that the clustering of cells into discrete populations is more diffuse in the tetO-2x set (Fig. 2, bottom row). Given these two points, the tetO-7x circuit variant might be expected to be more likely to yield exclusive decisions; however, comparison of individual response profiles reveals four exclusive response profiles (out of 48) in the tetO-2x set (Additional file 1: Figure S2) but none in the tetO-7x set (out of 25; Additional file 1: Figure S3).

Our results demonstrate that promoter architecture can impact decision-making behavior, and suggest the following mechanism as the driver of the differences in behavior between the two circuit variants. In the context of only activation or only suppression, one would expect an increase in the number of operator sites to increase the magnitude of the effect. However, when activation and inhibition are exerted simultaneously at a locus, one would expect a distribution in a population. For instance, if the promoter contains only one operator site, then this site can be in three possible states: unbound, bound by an activator, or bound by a repressor. An increase in the number of operator sites in the promoter leads to significantly more possible configurations, and appears to give rise to an averaging effect; in other words, the activity of the promoter as a function of activator and repressor levels would be expected to be more switch-like with 1–2 operator sites, but more graded with a larger number of operator sites. In the context of the CAA topology, the tetO-7x operator may be subject to more configurational fluctuations, and this behavior may prevent one side of the system from gaining an unassailable advantage over the other.

This analysis has broader implications for gene regulation: the number of operator sites can be an important variable in situations where contradictory actions are exerted at the same loci, which is likely true for a number of genes in higher organisms.

### Gene dosage modulates dynamic behaviors

The *T*_*A*_-*T*_*B*_ response profiles can be interpreted as comprising four populations: (low *T*_*A*_, low *T*_*B*_); (high *T*_*A*_, low *T*_*B*_); (low *T*_*A*_, high *T*_*B*_); and (high *T*_*A*_, high *T*_*B*_). If each population is either present or absent, then 16 potential response profiles (or phase portraits) are possible. The actual number is significantly higher given that the population centroids and the distribution of cells across the four populations can also both vary (see also Fig. 2 and Additional file 1: Figures S1-S3).

However, our experimental results suggest that individual clones from transformation reactions with different plasmids (e.g., 7x vs. 2x tetO operator sites) can yield highly similar response profiles, or phenotypes, suggesting that the basic CAA topology yields a limited number of response profile archetypes. To explore this further, we applied a clustering method to all response profiles.

Briefly, each clonal response profile was binned into an *n × n* grid, and a distance metric was computed for each pair of response profiles (see Methods). The distances were used as input into the partitioning around medoids (PAM) algorithm, which partitions data points into groups through iterative optimization [23], to obtain clusters of response profiles. Reasonable values for *n*, the number of bins for each of the two dimensions, and *k*, the number of clusters, were obtained empirically by varying both parameters, and analyzing silhouette scores of the resulting clusterings.

**Fig. 3:**
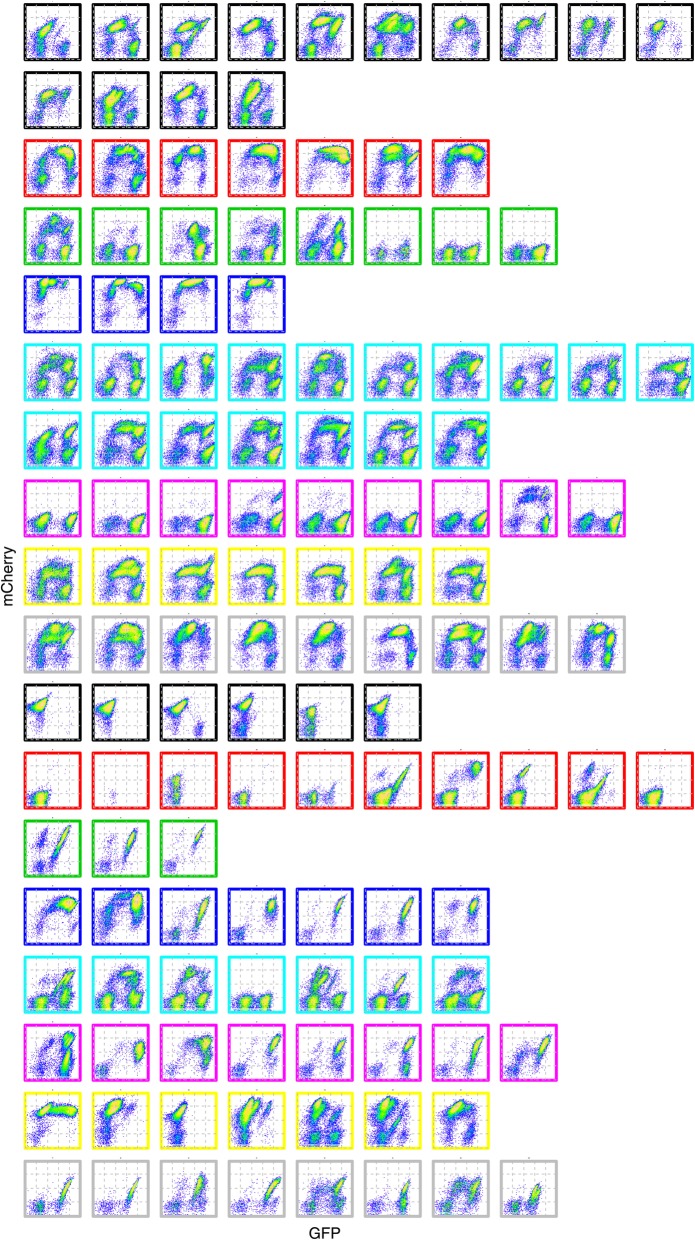
CAA topology yields a limited number of response profile archetypes. All response profiles obtained in the experiment were processed, binned, and clustered using the Manhattan distance metric and the PAM algorithm with k = 16. Different outline colors for individual response profiles denote the 16 different clusters. Flow cytometry data were captured at the 16-h time-point

Cluster analysis (Additional file 1: Figure S4) suggests that all response profiles can be appropriately partitioned into ~ 16 representative groups, a modest number, given the number of potential phase portraits (Fig. 3). At the same time, individual clones from the same transformation reaction, and hence having the same set of plasmids, can exhibit multiple phenotypes. Additionally, clones with different circuit components can yield markedly similar response profiles. Taken together, these results suggest that response profile diversity is significantly influenced by differences in gene dosage; in other words, different clones integrate the two plasmids in different copy numbers, and thus modify the dynamics of the overall system in a given cell.

To further explore this potential role of gene dosage, we developed a mathematical model that captures the behaviors exhibited by the family of circuits constructed for this study. The model comprises a pair of transcription factors, *A* and *B*, each of which promotes its own synthesis and inhibits the synthesis of the other. The current model differs from our previous models of the CAA topology [10, 21] in that it explicitly represents the inactive and active states of promoters for *A* and *B*. Additionally, the model represents mRNA and protein separately, allowing accounting of the different synthesis and degradation rates of these species.

The combination of these features allows the model to capture the property of transcriptional bursting, which has previously been shown to be important in generating bimodality in a simpler positive feedback circuit [24]. Starting with this general setup, we varied the model’s promoter counts to simulate different plasmid copy numbers, and varied other parameters to simulate strong versus weak repression.

**Fig. 4:**
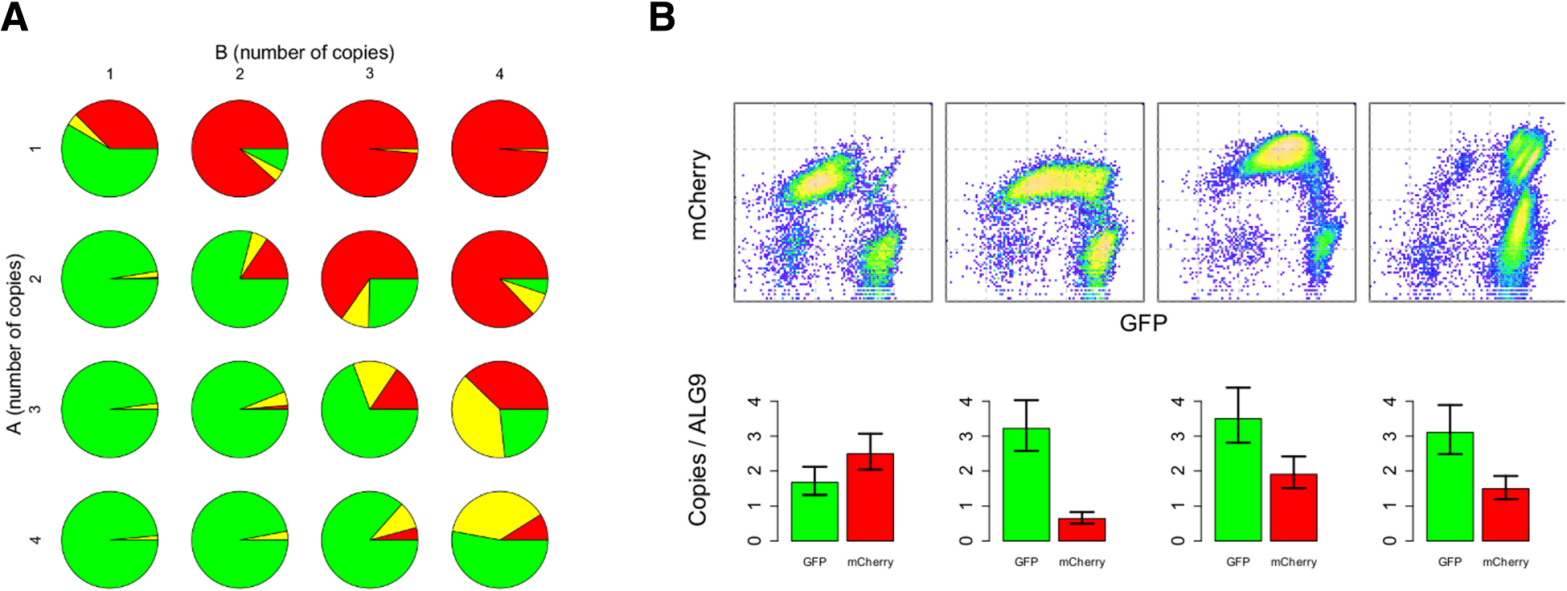
Gene copy-number can modulate CAA response profile. **a** Model simulations predict that gene copy number can skew response profiles. The CAA network topology was stochastically simulated under different circuit copy numbers for *A* and *B*. For each copy-number combination, 1000 simulations were performed, and the *A* and *B* levels at steady state were compiled to create a pie chart in which green denotes high expression for *A* and low expression for *B*, red denotes the reverse, and yellow denotes high expression for both *A* and *B*. **b** Selected strong repression tetO-2x clones were analyzed by flow cytometry to assess circuit expression, and by digital PCR to quantify the number of integrated copies of the two circuit plasmids. The GFP and mCherry genes were used as proxies for their respective plasmids. The native yeast gene ALG9 was used as an endogenous control. Error-bars represent 95% confidence intervals

Simulations reveal that our model can yield the general phenotypes exhibited by our circuits. Specifically, weak repression can lead to a larger proportion of the population with high levels of both *A* and *B*, as observed in our experiments. Comparison of results reveals another pattern that is consistent across simulations of different parameter combinations: higher promoter copy numbers yield increased proportions of the non-exclusive (high *A*, high *B*) population (Fig. 4a, Additional file 1: Figure S5).

In a CAA system with the opposing promoters present at one copy each, imbalances in basal expression of *A* and *B* can quickly arise and enable one transcription factor to gain an unassailable advantage by allowing it to support the activation of its own promoter, while suppressing the activity of the opposing promoter. However, this exclusivity between *A* and *B* is more difficult to obtain in an analogous system with multiple copies of both promoters. First, initial levels of *A* and *B* are more balanced due to the averaging effect of multiple promoter copies. Second, the probability that all promoters for *B* are repressed at a given point in time is by definition higher in a system with fewer promoter copies, given specific levels of *A* and *B*. Hence, as the number of promoter copies is increased, the system not only becomes less likely to generate a significant contrast between the expression levels of the two transcription factors, but also less likely to maintain that contrast.

To test this behavior experimentally, we selected four clones with differing GFP and mCherry response profiles from the tetO-2x set with strong repression, and we used digital PCR to quantify copy numbers of the two plasmids. Clones with fewer and balanced copy numbers of the two plasmids can yield robustly exclusive response profiles, but circuits with increased plasmid copies yield a larger proportion of cells expressing both transcription factors (Fig. 4b). These results suggest that a change in copy number does not merely yield trivial differences in phenotype, but can modulate the fundamental changes in dynamic behavior of the CAA circuit.

## Discussion

Our findings should provide greater insights into the regulatory mechanisms of cellular systems that naturally utilize the CAA topology for decision-making, including the differentiation of multipotent stem and progenitor cells that rely on this motif to achieve mutually exclusive cell fates [10, 12]. This study also highlights the complex interplay between promoter architecture and cellular response, which should motivate studies of other natural decision-making topologies in which the expression of key genes is modulated by both activators and repressors. With high copy numbers, it becomes difficult to yield exclusive response profiles, suggesting that a change in gene dosage through deletion or amplification, as is common in cancer [25], can not only affect targets directly downstream, but in the context of a dynamical system, can also deleteriously alter the energy landscape by introducing strong biases toward certain states or by introducing corrupt intermediate states.

This study should also further inform engineering design strategies for synthetic biology applications. The conceptual approach of part reuse at the protein level to achieve both orthogonal transcriptional specificities and tunable function (i.e., activation or strong/weak repression) can be further extended by leveraging more sophisticated programming of eukaryotic transcription factor function [26]. Additionally, since robust inducer-transcription factor systems are relatively scarce in synthetic biology, reuse can enable more complex networks to be constructed from the available components. Finally, the CAA topology represents a straightforward way to endow cells with mutually exclusive decision-making capabilities and our findings highlight specific design features – strong mutual repression, minimal number of operator sites, and low and balanced gene copy number – for its synthetic implementation.

## Conclusions

In this study, we used a synthetic biology approach to explore the plasticity of the response of a common decision-making topology, the CAA. We employed the engineering principle of reuse to construct this topology from a single core protein part, TetR, which was tuned through mutation to achieve two orthogonal specificities and through fusion partners to create the desired activating and repressive activities. We found that our synthetic CAA circuit is robust in yielding discrete population states, though the relative distribution of cells across these states can vary as a function of applied perturbations. We specifically examined perturbations to the CAA network that do not change its topological structure but that have the potential to influence its dynamic behavior, focusing on the strength of transcriptional repression, the number of operator sites, and gene dosage.

When the strength of transcriptional repression is attenuated, the negative coupling between the two transcriptional nodes is reduced, allowing both transcription factor abundances to rise and leading to a loss of transcriptional exclusivity. When the number of operator sites is increased in the CAA network, we unexpectedly found that exclusivity in decision-making was also hindered, due in part to the fact that both activating and repressive proteins act on the promoters, leading to an averaging effect that prevents either transcription factor from attaining a significantly greater concentration than its counterpart. Finally, we found both computationally and experimentally that gene dosage plays a critical role in balancing the abundance of network components, which in turn dictates whether the CAA topology can engender exclusive cell states. Our results demonstrate that perturbations that preserve the topology of a gene regulatory network can nonetheless significantly modulate response dynamics of the network.

## Methods

### Parts construction

Transcriptional activator genes were constructed as follows (with primer sequences listed in Additional file 1: Table S1). The tTA gene was amplified from the pUG6-tTA plasmid [27] (obtained from EUROSCARF, Accession P30385) using primers NASo065 and NASo055. This version of tTA has dimerization and operator-site specificities of type 0, as defined above. The yEGFP3 gene was amplified from pSP001 [28] with primers NASo057 and NASo058, and cloned into the tTA gene via BssHII digestion and ligation. This gene is referred to as tTA_0_ in the text.

To construct tTA_1_, PCR reactions were performed with pUG6-tTA as template. The first reaction was with primers NASo055 and NASo068; the second was with primers NASo067 and NASo070; and the third was with primers NASo069 and NASo065. Overlap PCR was performed on purified products from these three PCR reactions, and then outer primers NASo065 and NASo055 were used to amplify the resulting gene. The mCherry gene was amplified from plasmid eco062 [29] using primers NASo075 and NASo076, and cloned into the tTA_1_ gene via BssHII digestion and ligation.

Two types of transcriptional repressors were constructed, the TetR protein alone, or fused with the SSN6 repression domain. Construction of both types in the two specificities yields four separate genes. To construct TetR-only genes, primers NASo077 and NASo078 were used in PCR reactions with tTA_0_ and tTA_1_ separately as templates. To construct TetR-SSN6 fusions, part of the SSN6 gene was amplified from the *Saccharomyces cerevisiae* genome using primers NASo080 and NASo081. Next, TetR genes of the two specificities were separately amplified using primers NASo077 and NASo079. Purified products from the preceding two PCR reactions were used in an overlap PCR reaction, and further amplified with outer primers NASo077 and NASo081.

### Plasmid construction

The bi-directional promoter system was constructed as follows. First, two separate PCR reactions were performed with plasmid eco008 [29] as template and primer pairs NASo001/NASo002 and NASo003/NASo004. Purified products from these reactions were used in an overlap PCR reaction and outer primers NASo001 and NASo004 were used to further amplify the product. This product was digested with XhoI and BamHI, and ligated into plasmid eco008 to construct plasmid pNAS001.1.

Primers NASo036 and NASo037 were used to amplify the CMV promoter and the ADH1 terminator from pNAS001.1. The product was digested with EcoRI and AvrII, and ligated into plasmid pNAS001.1. Next, the CYC1 TATA minimal promoter was amplified from plasmid pNAS001.1 with primers NASo059 and NASo060 and the product was cloned into the new plasmid via AvrII and XhoI enzymes. This plasmid establishes the following promoter architecture. First, the space between AvrII and ClaI sites is used to conveniently insert the desired operator: canonical tetO-2x, tetO-4C5G-2x, canonical tetO-7x, or tetO-4C5G-7x. On both the 5′ and 3′ ends of the operator space are copies of the minimal CYC1 TATA promoters. Following the CYC1 TATA sequence on the 3′ side are BamHI and NotI restriction sites, followed by the CYC1 terminator sequence. Similarly, following the CYC1 TATA sequence on the 5′ side are AflII and XhoI restriction sites, followed by the ADH1 terminator. The resulting sequence was cloned into the HIS3 backbone of pERT252 via restriction digestion and ligation, creating two plasmids with different auxotrophic selection markers (URA3 and HIS3).

The tTA_0_ and tTA_1_ activator genes (together with the respective GFP or mCherry fusion) were cloned in via the BamHI and NotI sites, while the repressor genes, tTS_0_ and tTS_1_, were cloned in via the AflII and XhoI sites. Different tetO operator sequences were either purchased or constructed via PCR. These sequences were cloned into the plasmids via AvrII and ClaI cloning.

The base plasmid for the circuit series was constructed as follows. Plasmid pNAS001.1 was digested with AvrII and ClaI, and the tetO-2x sequence was ligated into it to construct plasmid pNAS004.1. Similarly, the tetO-4C5G-2x sequence was ligated into the pNAS001.1 plasmid (digested with AvrII and ClaI). Primers NASo099 and NASo100 were used to amplify the kanamycin resistance gene (KanR) from the pUG6-tTA plasmid, and this was ligated into the two plasmids. This yields two constructs with KanR driven by tetO-2x and tetO-4C5G-2x, respectively.

Primers NASo112 and NASo113 were used to amplify the KanR cassette from the plasmid containing the tetO-2x sequence, and the resulting product was cloned into plasmid eco007 [29] via XmaI and XhoI to place the KanR tetO-2x cassette in a LEU2 auxotrophic marker background. Primers NASo001 and NASo041 were used to amplify from the plasmid containing KanR driven by tetO-4C5G-2x sequence, and the resulting product was ligated into the new plasmid containing KanR driven by tetO-4C5G-2x sequence and a LEU2 background via XhoI and MluI cloning. The ligated plasmid was named pNAS135.

The pNAS135 plasmid consists of two copies of the KanR gene, driven by the tetO-2x and tetO-4C5G-2x operators. In the context of the circuit, the KanR protein is expressed when either or both of tTA_0_ and tTA_1_ are present in sufficient quantities. Hence, addition of G418 to the culture medium will inhibit growth of cells not expressing significant levels of both tTA proteins. This mechanism facilitates additional perturbations.

Reagents for cloning were obtained from the following sources: restriction enzymes from New England Biolabs, Phusion polymerase from Finnzymes, and custom oligonucleotides from Integrated DNA Technologies.

### Yeast transformation

The NASy001 strain was constructed by transforming BMA64-1A [30] yeast cells with the pNAS135 plasmid, and PCR-screening to identify a single-integrand. All CAA circuit plasmids were transformed into NASy001. Circuit plasmids for the two opposing sides in each circuit variant were transformed simultaneously into NASy001 via the LiAc/SS carrier DNA/PEG protocol [31]. Transformation reaction mixes were plated onto agar plates containing synthetic selective medium, as well as 5 g/mL anhydrotetracycline (aTc, Sigma) to inhibit activation of the circuit during post-transformation growth. aTc was used instead of doxycycline for this step since it has a longer half-life (the post-transformation growth period lasts 48–72 h). Individual clones were picked for all subsequent analyses by flow cytometry or microscopy. Transformation reagents were obtained from Sigma.

### Yeast cultures

After obtaining transformants for the different variations of the CAA circuit, we performed a global survey of behavior. For each circuit variant, several individual clones were separately inoculated into selective liquid medium, supplemented with 5 μg/mL doxycycline. In the presence of high levels of doxycycline, the TetR proteins cannot bind to DNA, and hence their activation or repression activity is abrogated, resulting in complete inhibition of the circuit. After overnight growth in liquid medium, the cultures were diluted into fresh medium containing doxycycline, and incubated for another 6–8 h to enable cells to leave the lag phase. Subsequently, the cultures were centrifuged, washed twice with PBS, and resuspended in fresh medium lacking doxycycline to allow expression of the circuit. Since the activator for Set 0 is fused to GFP, and the activator for Set 1 is fused to mCherry, the levels of the transcription factors can be tracked by assaying for GFP and mCherry expression.

### Flow cytometry data analysis

GFP and mCherry expression was assessed by flow cytometry using a BD LSRFortessa instrument (BD Biosciences) equipped with 488-nm and 561-nm lasers for excitation and 530/30 and 610/20 emission filters, respectively. Data files from the instrument were imported into the FlowJo software, and the FSC-H, FSC-W, FSC-A, and SSC-A parameters were used to exclude events not corresponding to individual yeast cells. After gating, custom R programs were used to pool flow cytometry data and trim outlier events. Subsequently, each response profile was binned into an *n × n* grid, with *n* bins each for GFP and mCherry. For plotting all figures, *n* = 100 was used while for clustering of response profiles, *n* = 10 was used.

For cluster analysis of flow cytometry data, a distance metric was computed between each pair of binned response profiles. Each binned response profile was treated as a vector, and the Manhattan distance metric [32] was used to compute the dissimilarity. Masks for aggregated flow cytometry response profiles were created by first processing and binning each response profile as described above, and then computing an average over all of the response profiles. The data were normalized before averaging such that each response profile carries the same weight.

### Plasmid copy-number estimation via digital PCR

For selected clones, the numbers of integrated copies of the two plasmids were estimated using digital PCR on an OpenArray Real-Time PCR System (Life Technologies). Briefly, clones were inoculated and grown in selective medium containing high levels of doxycyline to suppress circuit expression. Saturated cultures were then processed to extract purified genomic DNA. The genomic DNA was digested overnight at 37 °C with SacI-HF (New England Biolabs). The digested genomic DNA was then appropriately diluted and used as template in separate digital PCR reactions along with TaqMan probes (Life Technologies) targeting the GFP, mCherry, or ALG9 genes. For quantifying the number of integrated copies, both GFP and mCherry are considered proxies of their respective plasmids, with the native yeast gene ALG9 being an endogenous control.

### Live-cell imaging

GFP and mCherry expression dynamics of individual cells were monitored using a protocol modified from the literature [33]. Cells from individual clones were grown in selective medium containing doxycycyline (Sigma) to suppress circuit expression, subsequently washed to remove doxycycline, and then transferred to agar pads containing SC medium. After a few minutes, the pads were inverted and placed in a microscopy dish. A Deltavision Deconvolution Station (consisting of an Olympus IX70 inverted microscope, a Photometrics CoolSnap HQ high-resolution CCD camera, and a motorized stage) was used to acquire images at 15-min intervals.

### Mathematical modeling

A mathematical model of the CAA circuit was constructed similarly to our previous models [10, 21], but with the explicit inclusion of active and inactive promoter states and the separate accounting of mRNA and protein states to allow different synthesis and degradation rates of these species. Full details of the model are provided in Additional file 1: Note 1.

## Additional files

Additional file 1: Supporting figures, table, video legends, and model details. (PDF 2765 kb)

Additional file 2: Video of strong inhibition in the CAA circuit. (AVI 51461 kb)

Additional file 3: Video of weak inhibition in the CAA circuit. (AVI 51461 kb)
