# Supplementary material for: Variable cellular decision-making behavior in a constant synthetic network topology
Source: BMC Bioinformatics. 2019 May 14;20:237. doi: 10.1186/s12859-019-2866-6 (PMC6515661; doi:10.1186/s12859-019-2866-6)
Supplement: Supplementary file 1 — Supporting figures, table, video legends, and model details. (PDF 2765 kb) [file 12859_2019_2866_MOESM1_ESM.pdf]

Supporting Information for:

Variable cellular decision-making behavior in a constant synthetic network topology

Najaf A. Shah[1] and Casim A. Sarkar[2,*]

[1]Genomics and Computational Biology Graduate Group, Perelman School of Medicine, University of Pennsylvania, Philadelphia, PA

[2]Department of Biomedical Engineering, College of Science and Engineering, University of Minnesota, Minneapolis, MN

[*]Corresponding author:

Casim A. Sarkar

csarkar@umn.edu

Figure S1: Response profiles for the CAA circuit with tetO-2x and weak repression.

GFP and mCherry response profiles for different clones of CAA circuits with tetO-2x operators and weak repression. Response profiles were processed and binned as described in the text. All profiles are on log-scales for GFP (x-axis) and mCherry (y-axis). For each axis, the distance between two dashed lines denotes a ten-fold change in expression.

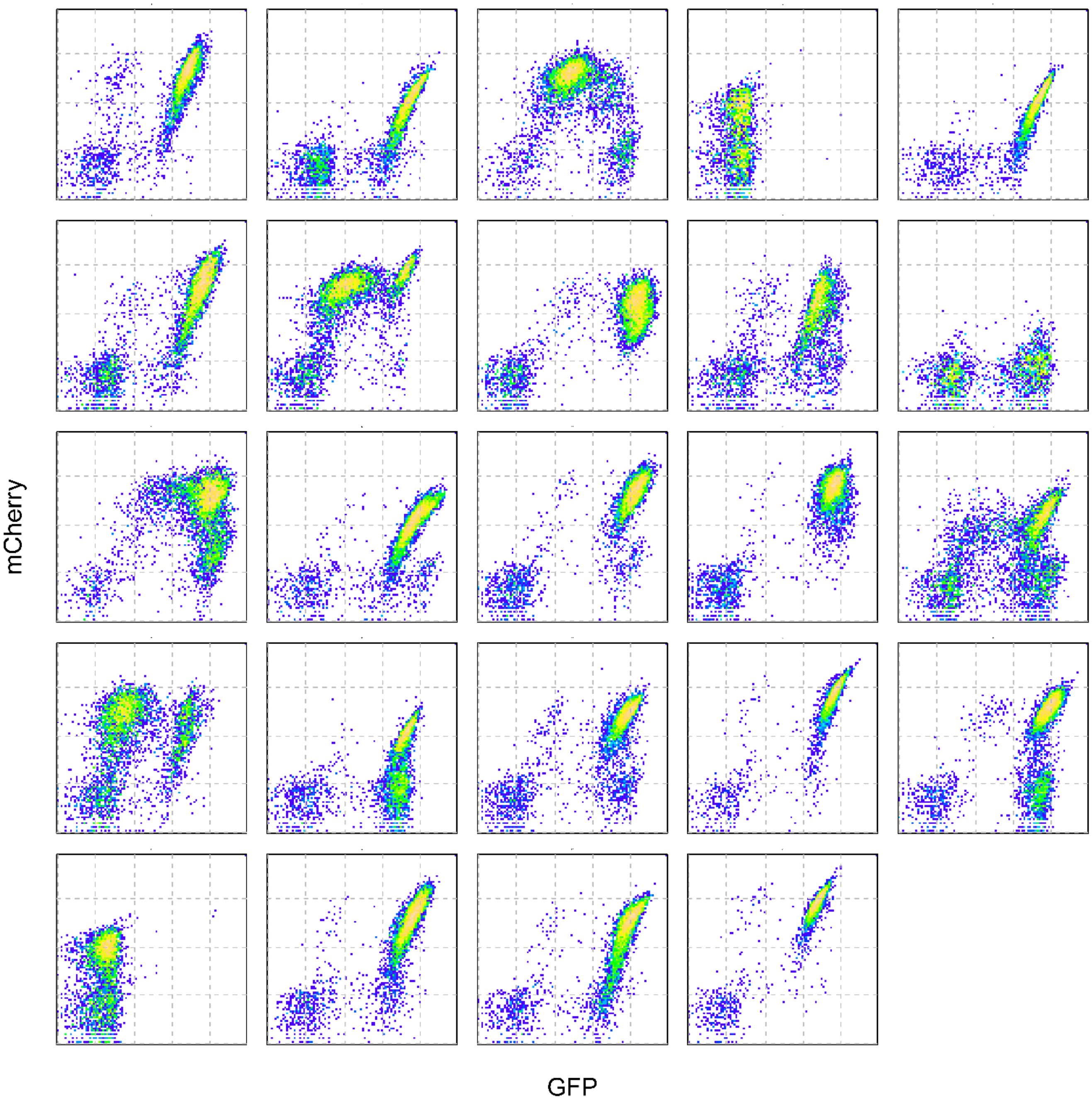

Figure S1 (cont.)

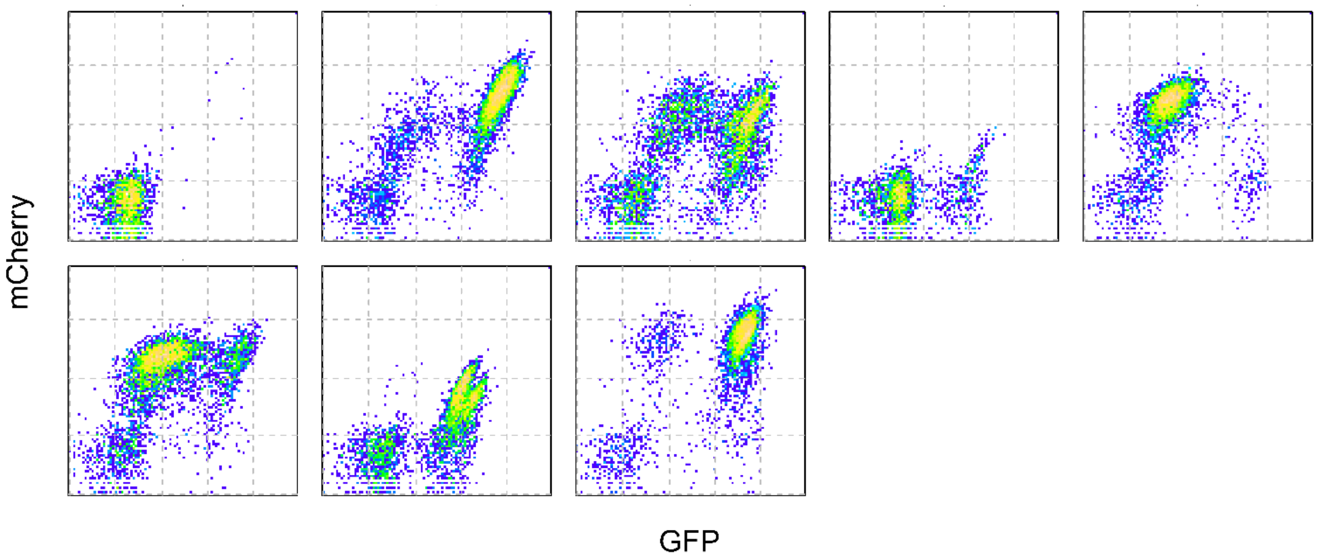

Figure S2: Response profiles for the CAA circuit with tetO-2x and strong repression.

GFP and mCherry response profiles for different clones of CAA circuits with tetO-2x operators and strong repression using SSN6 domains. Response profiles were processed and binned as described in the text. All profiles are on log-scales for GFP (x-axis) and mCherry (y-axis). For each axis, the distance between two dashed lines denotes a ten-fold change in expression.

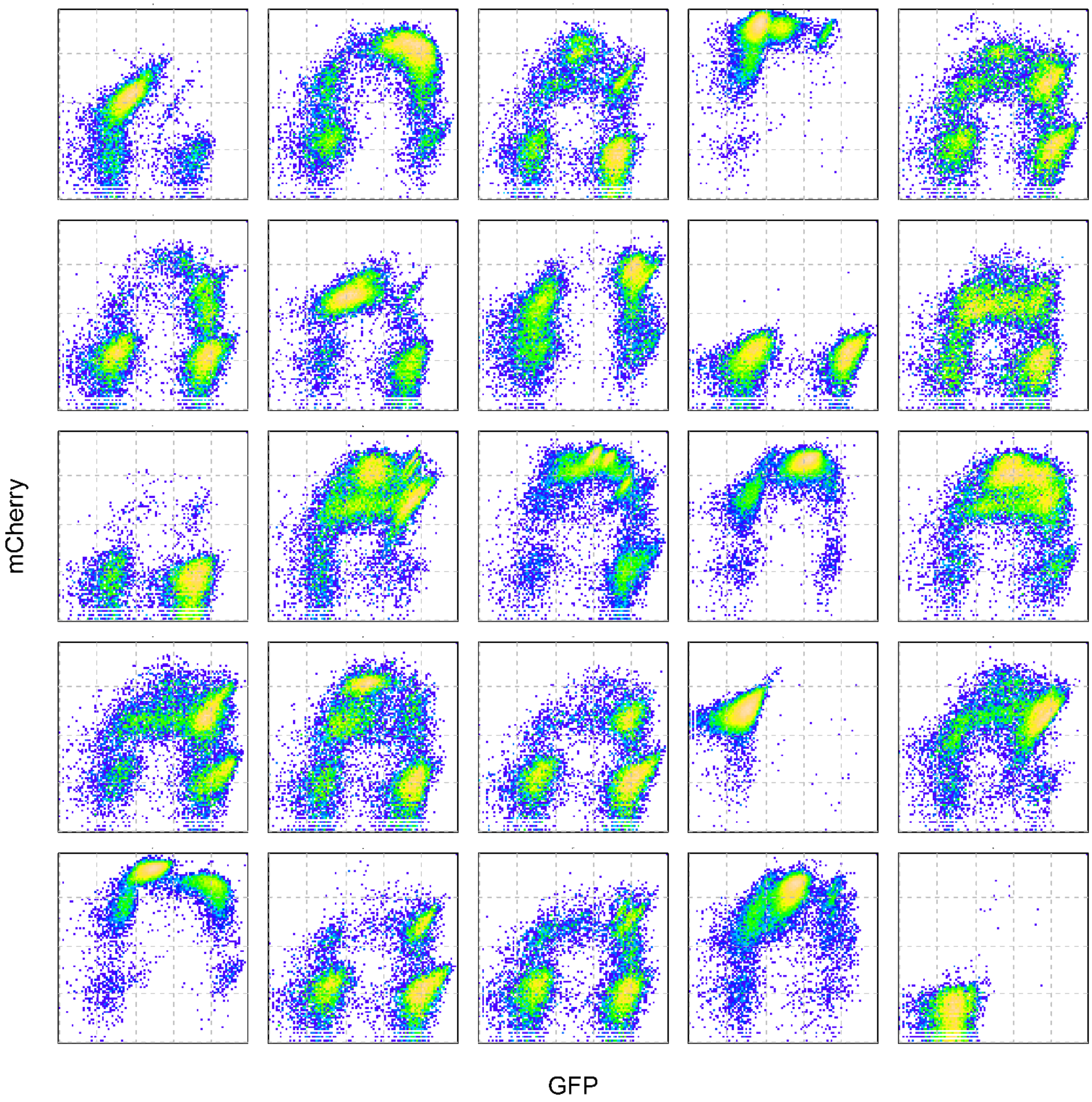

Figure S2 (cont.)

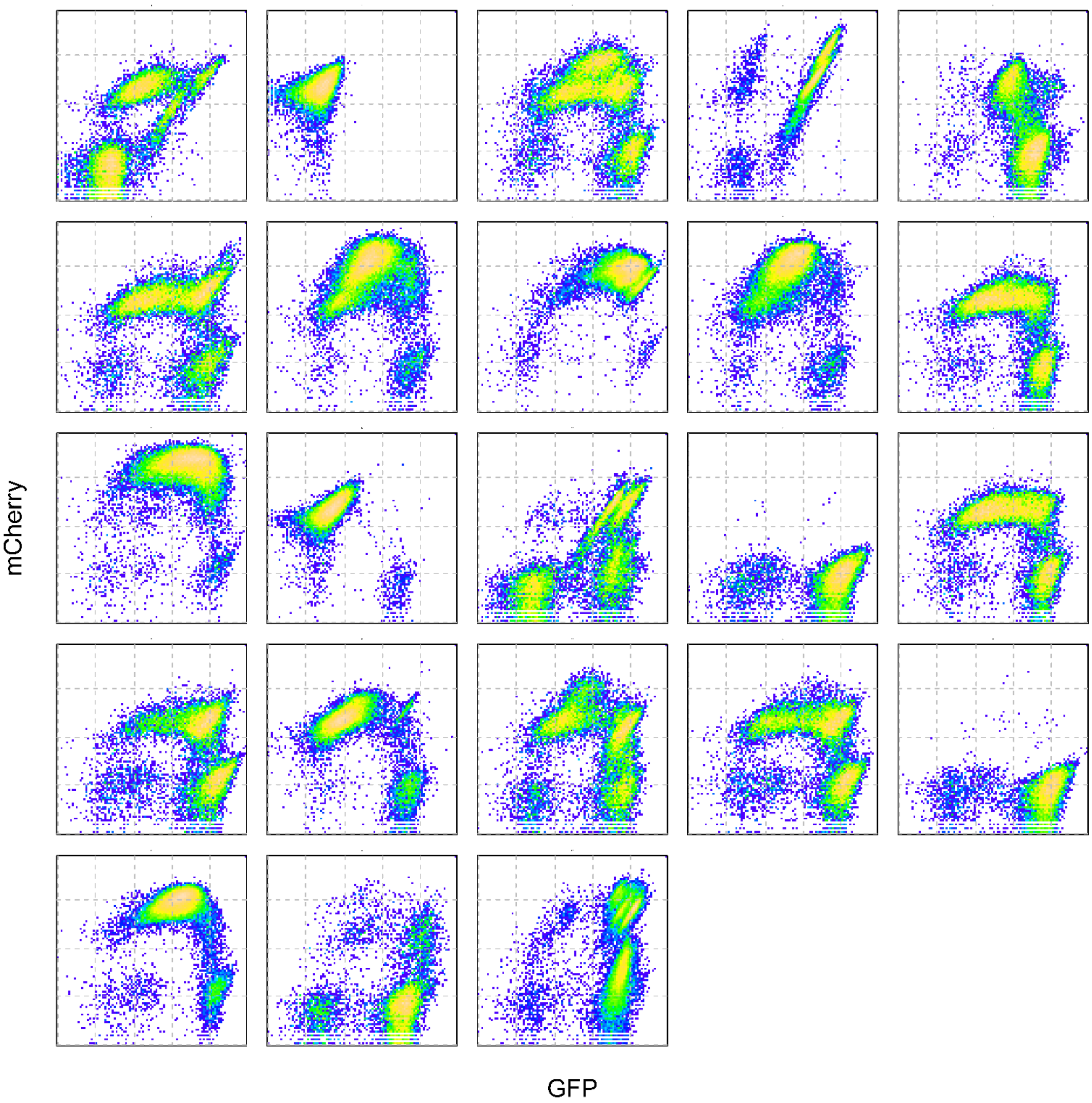

Figure S3: Response profiles for the CAA circuit with tetO-7x and strong repression.

GFP and mCherry response profiles for different clones of CAA circuits with tetO-7x operators and strong repression. Response profiles were processed and binned as described in the text. All profiles are on log-scales for GFP (x-axis) and mCherry (y-axis). For each axis, the distance between two dashed lines denotes a ten-fold change in expression.

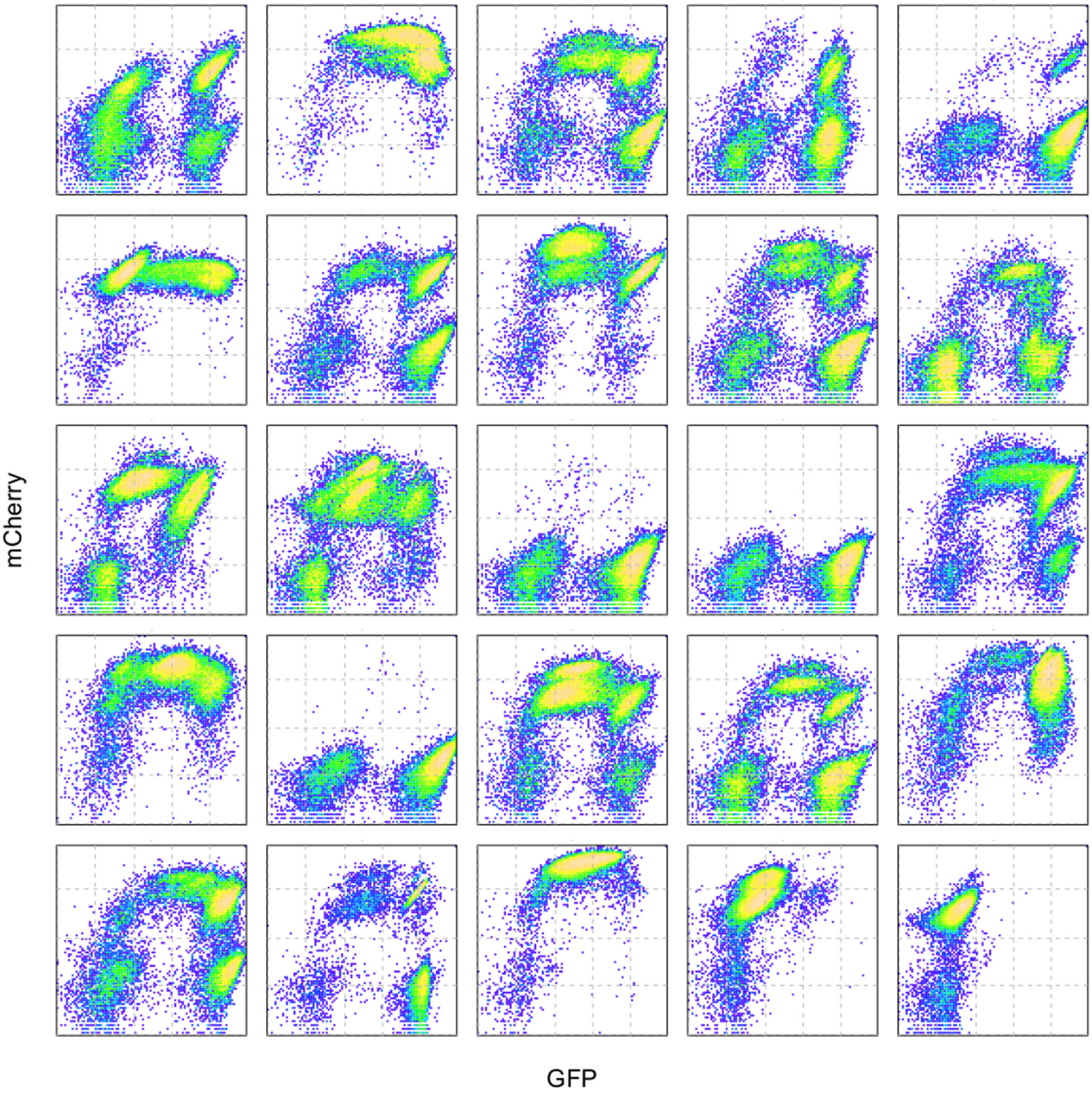

Figure S4: Clustering of response profiles.

All response profiles obtained in the experiment were processed, binned, and clustered using the Manhattan distance metric and the partitioning around medoids (PAM) algorithm with varying *k*. Median silhouette values were computed for clustering runs with varying *k*.

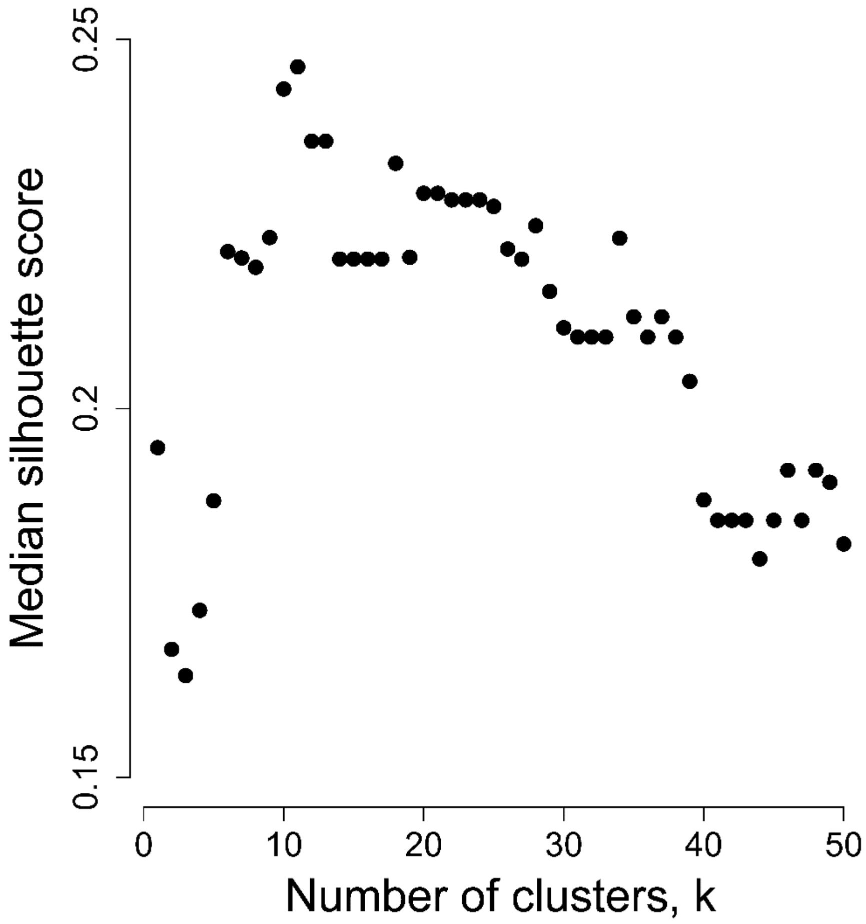

Figure S5: Simulation of CAA topology with weak repression under different copy numbers of *A* and *B*.

CAA network topology under weak repression was stochastically simulated under different circuit copy numbers for *A* and *B*. For each copy-number combination, 1000 simulations were performed, and the *A* and *B* levels at steady state were compiled to create a pie chart in which green denotes high expression for *A* and low expression for *B*, red denotes the reverse, and yellow denotes high expression for both *A* and *B*.

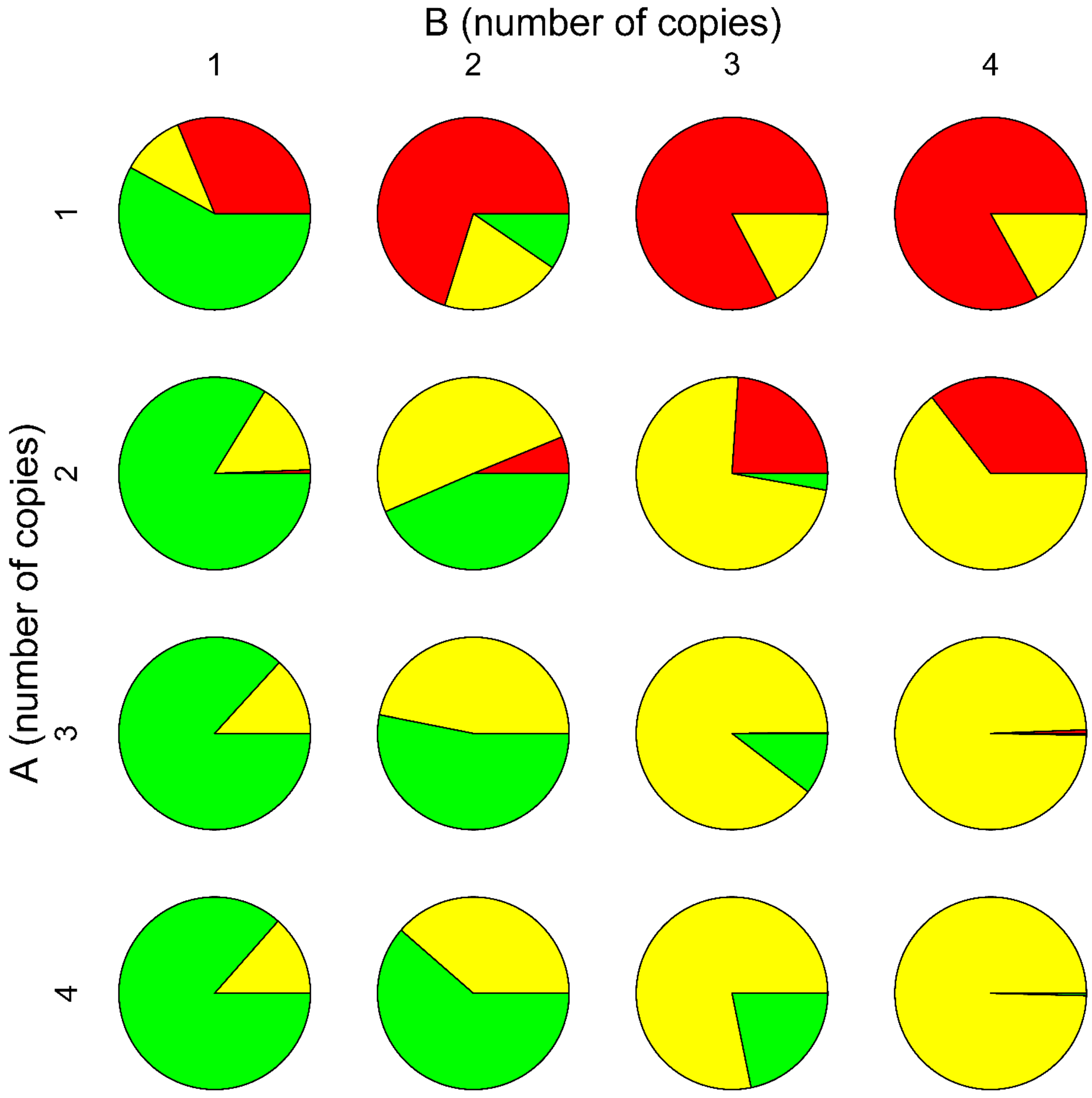

Table S1: Primer sequences used for construction of synthetic circuits.

NASo001	AAG CTC CTC GAG TAA TTC GCG C
NASo002	ATC GAT GGC GCC CCT AGG AAC TCG ATC GAG GAA TTG ATC TGC CGG TAG AGG TG
NASo003	CCT AGG GGC GCC ATC GAT AAA GTC GAG CTC GGT ACC CTA TGG CAT GCA TGT GC
NASo004	AGA CAT GGA TCC CCC GAA TTG
NASo036	AAT TCG GGG AAT TCG ATC TGC CGG TAG AGG TGT GG
NASo037	AAT TCG GGG GAT CCA TCG ATG GCG CCC CTA GGA TGA ATT AAT TCG GGC CGC G
NASo055	TAA TCA GTG GAT CCA TGT CTA GAT TAG ATA AAA GTA AAG TGA
NASo058	AAA CTA GTG CGC GCC TTT GTA CAA TTC ATC CAT ACC ATG G
NASo059	ACT GAT TAC CTA GGT ATG GCA TGC ATG TGC TCT GT
NASo060	CCC GAA TTC TCG AGA TCA GTC TTA AGA TCC CCC GAA TTG ATC CGG T
NASo065	ACT GAT TAG CGG CCG CCT ACC CAC CGT ACT CGT C
NASo067	GCC CAG AAG CTA GGT TTT GCG CAG AAA ACA TTG TAT TGG CAT
NASo069	CCA GCC TTC TTA CAC GGC CTT GAA TCG CTC ATA TGC GGA TTT GAA AAA CAA CTT
NASo070	AAG TTG TTT TTC AAA TCC GCA TAT GAG CGA TTC AAG GCC GTG TAA GAA GGC TGG
NASo075	CGA TAG GCG CGC TGG TTT CCA AGG GTG AAG AAG
NASo076	CGA TAG GCG CGC CCT TGT ATA ATT CGT CCA TAC C
NASo077	CGA TAG CTT AAG ATG TCT AGA TTA GAT AAA AGT AAA GTG
NASo078	CTA TCG CTC GAG TTA CCC ACT TTC ACA TTT AAG TTG T
NASo079	AGA TCC ACT TTC ACA TTT AAG TTG
NASo080	CAA CTT AAA TGT GAA AGT GGA TCT ATG AAT CCG GGC GGT GAA C
NASo081	AAT GTC GCT CGA GTT TAG TCG TCG TAG TTT TCA TC

Supporting Video Legends

Video S1: Strong inhibition in the CAA circuit can yield exclusivity and memory.

Cells from a clone from the tetO-2x, strong repression set were grown in selective medium containing high doxycycline to suppress circuit expression, were subsequently washed to remove doxycycline, and then transferred to an agar pad for time-lapse microscopy. Images show that under strong mutual inhibition, the CAA circuit can yield exclusive decisions with memory, in that cells that decide to commit to a state remain committed.

Video S2: Weak inhibition in the CAA circuit does not yield exclusivity.

Cells from a clone from the tetO-2x, weak repression set were grown in selective medium containing high doxycycline to suppress circuit expression, were subsequently washed to remove doxycycline, and then transferred to an agar pad for time-lapse microscopy. Images show that under weak mutual inhibition, the CAA circuit does not yield exclusive decisions, in that most cells express the transcription factors from both states at high levels.

Supporting Note 1: Model reactions, equations, and parameters.

Systems model for the CAA circuit.

Number	Reaction	Description	Rate
0	$P_A \longrightarrow P_A^*$	Activation of promoter A	$\left(k_0 + \frac{k_1}{1+\left(\frac{k_2(1+k_{32}B)}{A}\right)^h}\right) P_A$
1	$P_B \longrightarrow P_B^*$	Activation of promoter B	$\left(k_3 + \frac{k_4}{1+\left(\frac{k_5(1+k_{33}A)}{B}\right)^h}\right) P_B$
2	$P_A^* \longrightarrow P_A$	Inactivation of promoter A	$\left(k_6 + \frac{k_7}{1+(\frac{k_8}{B})^h}\right) P_A^*$
3	$P_B^* \longrightarrow P_B$	Inactivation of promoter B	$\left(k_9 + \frac{k_{10}}{1+(\frac{k_{11}}{A})^h}\right) P_B^*$
4	$P_A^* \longrightarrow P_A^* + M_A$	Synthesis of mRNA A	$k_{12}P_A^*$
5	$P_B^* \longrightarrow P_B^* + M_B$	Synthesis of mRNA B	$k_{13}P_B^*$
6	$M_A \longrightarrow \emptyset$	Degradation of mRNA A	$k_{14}M_A$
7	$M_B \longrightarrow \emptyset$	Degradation of mRNA B	$k_{15}M_B$
8	$M_A \longrightarrow M_A + A$	Synthesis of A	$k_{16}M_A$
9	$M_B \longrightarrow M_B + B$	Synthesis of B	$k_{17}M_B$
10	$A \longrightarrow \emptyset$	Degradation of A	$k_{18}A$
11	$B \longrightarrow \emptyset$	Degradation of B	$k_{19}B$
12	$P_A \longrightarrow P_A'$	Repression of promoter A	$\left(k_{20} + \frac{k_{21}}{1+\left(\frac{k_{22}(1+k_{33}A)}{B}\right)^h}\right) P_A$
13	$P_B \longrightarrow P_B'$	Repression of promoter B	$\left(k_{23} + \frac{k_{24}}{1+\left(\frac{k_{25}(1+k_{32}B)}{A}\right)^h}\right) P_B$
14	$P_A' \longrightarrow P_A$	De-repression of promoter A	$\left(k_{26} + \frac{k_{27}}{1+\left(\frac{k_{28}(1+k_{32}B)}{A}\right)^h}\right) P_A'$
15	$P_B' \longrightarrow P_B$	De-repression of promoter B	$\left(k_{29} + \frac{k_{30}}{1+\left(\frac{k_{31}(1+k_{33}A)}{B}\right)^h}\right) P_B'$

Number	Species	Description
0	P_A	Promoter A, inactive
1	P_B	Promoter B, inactive
2	P_A^*	Promoter A, active
3	P_B^*	Promoter B, active
4	M_A	mRNA A
5	M_B	mRNA B
6	A	Transcription factor A
7	B	Transcription factor B
8	P_A'	Promoter A, repressed
9	P_B'	Promoter B, repressed

Symbol	Description	Value
k_0	Basal activation of Promoter A	0.09
k_1	Maximal activation rate of Promoter A by A	1
k_2	A-level at which A-mediated activation of Promoter A is half-maximal	8500
k_3	Basal activation of Promoter B	0.09
k_4	Maximal activation rate of Promoter B by B	1
k_5	B-level at which B-mediated activation of Promoter B is half-maximal	12750
k_6	Basal inactivation of Promoter A	85
k_7	Maximal inactivation rate of Promoter A by B	0
k_8	B-level at which B-mediated inactivation of Promoter A is half-maximal	8500
k_9	Basal inactivation of Promoter B	85
k_{10}	Maximal inactivation rate of Promoter B by A	0
k_{11}	A-level at which A-mediated inactivation of Promoter B is half-maximal	12750
k_{12}	Synthesis rate of mRNA A	500
k_{13}	Synthesis rate of mRNA B	500
k_{14}	Degradation of mRNA A	1
k_{15}	Degradation of mRNA B	1
k_{16}	Synthesis rate of A	577.5
k_{17}	Synthesis rate of B	577.5
k_{18}	Degradation of A	0.25
k_{19}	Degradation of B	0.25
k_{20}	Basal repression of Promoter A	0
k_{21}	Maximal basal repression rate of Promoter A by B	0.15 or 0
k_{22}	B-level at which B-mediated repression of Promoter A is half-maximal	8500
k_{23}	Basal repression of Promoter B	0
k_{24}	Maximal repression rate of Promoter B by A	0.15 or 0
k_{25}	A-level at which A-mediated repression of Promoter B is half-maximal	12750
k_{26}	Basal de-repression of Promoter A	0.01
k_{27}	Maximal basal de-repression rate of Promoter A by A	1
k_{28}	A-level at which A-mediated de-repression of Promoter A is half-maximal	8500
k_{29}	Basal de-repression of Promoter B	0.01
k_{30}	Maximal de-repression rate of Promoter B by B	1
k_{31}	B-level at which B-mediated de-repression of Promoter B is half-maximal	12750
k_{32}	Competitive inhibition of Promoter A by B	1/85000
k_{33}	Competitive inhibition by Promoter B by A	1/85000
h	Hill coefficient	2
